# Medical Jurisprudence

**Published:** 1892-01

**Authors:** Henry A. Riley

**Affiliations:** New York


### MEDICAL JURISPRUDENCE.

CONDUCTED BY HENRY A. RILEY, A. B., LL. B., New York.

### CORONERS' JURIES AND THE PARK PLACE DISASTER.

THE terrible accident in New York City not long since, by which some sixty-four persons lost their lives through the collapse of a building, where heavy printing machinery was stored, has served to call to mind not only the defects of the building laws, which permit the overloading of buildings where it may be scores of persons are employed, but also the peculiar workings of the system of coroners' inquests. It was supposed by the public that such an appalling disaster would bring home to some one the charge of criminal negligence in not looking more carefully after the safety of human lives.

The coroner's jury failed, however, to find anyone responsible, and laid the whole fault upon the defects of the law, which did not provide for the services of a sufficient number of inspectors to properly examine buildings where heavy machinery was stored. The grand jury has also just taken the same view of the matter, and consequently there will be no prosecutions whatever. The latter body makes the following recommendation for legislation, which is certainly timely: "The placing within any building of a greater weight than the building is capable of safely supporting, should be made a felony, and the fact of such overloading being established, the owner, as well as the occupant of the building, or the part thereof overloaded, should be *prima facie* guilty."

---

### COURTS DIFFER IN REGARD TO INHALING GAS.

The United States Circuit Court for Illinois differs with the Court of Appeals of this State on the question of the liability of accident insurance companies for death by inhaling gas.

In a recent case in Chicago, a person was found dead at a hotel with the gas turned on in the room. There was no visible sign of violence or external injury on the body, and nothing to show whether it was a case of suicide or an accident. The limbs and features were not distorted, and the victim was lying naturally on his side in bed. He held a policy of accident insurance in the Travelers' Insurance Co., which expressly relieved the company from all liability when death occurred by inhaling gas. The court held that the company need not pay the policy, and quoted the adverse opinion in a case in the New York Court of Appeals, where it was said: "In expressing the intention not to be liable for death from inhaling gas, can only be understood to mean a voluntary and intelligent act by the insured, and not an involuntary and unconscious act. Read in that sense and in the light of the context, these words may be interpreted as having reference to medical or surgical treatment in which *ex vi termini* would be included the dentists' work or a suicidal purpose."

The Illinois court comments on this decision and says: "The reasoning by which that court reached its conclusion is not satisfactory to my mind. The language of the policy is so clear as not to require any construction. The words are unequivocal that the defendant does not insure against death caused by inhaling gas.

There is nothing in the terms of the policy intimating or suggesting that the inhalation of gas must be voluntary or involuntary in order to exempt defendant from liability. That the defendant had the right to so limit its liability, there can be no doubt."

### MALPRACTICE AND MORPHINE.

A South Dakota court has now before it a suit for money damages against a physician who, it is alleged, has by continuous hypodermic injections of morphine, rendered a patient a mental and physical wreck. The action is brought by the wife, who states that she has been deprived of her support through the improper treatment of the physician.

This is a novel case, and yet foreshadows what may be a long line of malpractice suits, for there is little doubt that the morphine habit is growing, and that physicians are sometimes responsible for the sad results.

### A NOVEL PUNISHMENT FOR TRESPASSING.

An English surgeon, not long since, found two boys trespassing in his garden, and, by way of punishment, painted their faces with lunar caustic, producing the representation of a moustache and imperial. When the boys got home, their faces were dressed with oil, but they complained very much of the pain, and the next day one of them was sent home from his place of work because the employer objected to his facial adornment. The parents afterwards brought an action against the physician, and medical evidence was given as to the nature of the injuries.

The court fined the surgeon five shillings and costs.

### A NEBRASKA MALPRACTICE CASE.

In a Nebraska court recently a physician was sued for $10,000 for alleged malpractice. The patient and her husband claimed that the physician had performed an abortion, while he asserted that he had removed a tumor.

The jury granted a verdict for $97.50, which was the exact amount recovered by the physician in a previous action for medical services.

